# Perceptions of exercise benefits and barriers: the influence on physical activity behaviour in individuals undergoing haemodialysis and peritoneal dialysis

**DOI:** 10.1007/s40620-021-01024-y

**Published:** 2021-03-26

**Authors:** Courtney J. Lightfoot, Thomas J. Wilkinson, Yan Song, James O. Burton, Alice C. Smith

**Affiliations:** 1 Leicester Kidney Lifestyle Team, Department of Health Sciences, University of Leicester, Leicester, LE17RH UK; 2 Leicester NIHR Biomedical Research Centre, Leicester, UK; 3 Medical School, Nantong University, Nantong, China; 4 Department of Cardiovascular Sciences, University of Leicester, Leicester, UK

**Keywords:** Dialysis, Exercise, Barriers, Benefits, Physical activity

## Abstract

**Background:**

Despite growing evidence about the benefits of physical activity and exercise in patients receiving dialysis, physical inactivity is highly prevalent. This may be due to uncertainty and lack of appropriate guidance about exercise, or driven by the relative barriers and benefits that patients perceive. Understanding these perceptions in dialysis patients may inform interventions aimed to increase exercise participation.

**Methods:**

Perceived benefits and barriers to exercise were measured by the ‘Dialysis Patient-perceived Exercise Benefits and Barriers Scale’ (DPEBBS). Self-reported physical activity status was assessed by the ‘General Practice Physical Activity Questionnaire’. Barriers and benefits to exercise were classed as binary variables (i.e. yes and no). Frequency analyses and chi-squared tests were conducted to compare the differences perceived by people on haemodialysis (HD) and peritoneal dialysis (PD). Binominal logistical regression was performed to determine which perceived barriers and benefits had the biggest impact on physical activity status.

**Results:**

One thousand twenty-two HD and 124 PD patients completed the DPEBBS. A greater proportion of HD than PD patients reported ‘reduces body pain’ (P = 0.013), ‘delays decline in body function’ (P = 0.01), and ‘improves quality of life’ (P = 0.033) as benefits of exercise. No differences in barriers were observed. Tiredness was the most reported barrier to exercise. Patients who perceived ‘other comorbidities’ (OR 3.389, P < 0.001) or ‘burden of family’ (OR 3.168, P < 0.001) as barriers were 3 times more likely to be inactive.

**Conclusions:**

Dialysis patients perceive several barriers which may prevent them from engaging in physical activity. Addressing these barriers may be key to increasing participation in physical activity and exercise.

**Graphic abstract:**

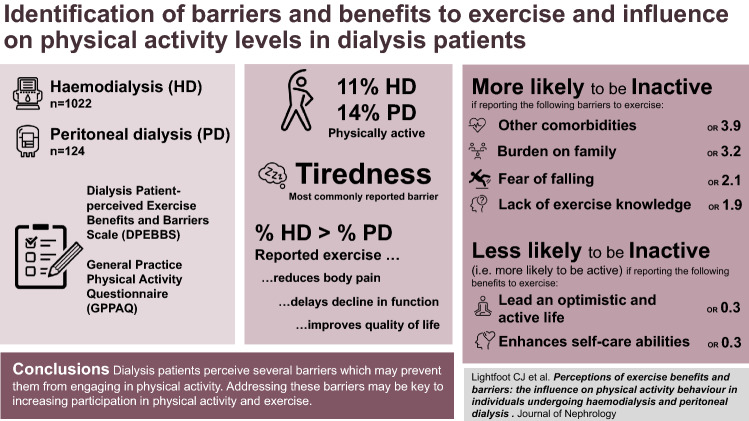

**Supplementary Information:**

The online version contains supplementary material available at 10.1007/s40620-021-01024-y.

## Introduction

Individuals undergoing dialysis are considerably less physically active than both the general population and those with milder stages of kidney disease. Despite the myriad of benefits including, but not limited to, improved cardiovascular function, muscular health, physical performance, and dialysis adequacy [1, 2], only ~ 6% of haemodialysis (HD) and 8% of peritoneal dialysis (PD) patients are sufficiently ‘active’ for health, of whom, only 5% and 6% of HD and PD patients, respectively, engaged in physical exercise for > 1 h/week [3].

Part of the physical inactivity paradigm may be the lack of promotion of physical activity and exercise by healthcare professionals. The reasons for this are numerous, including a concern for safety and uncertainty about the most appropriate exercise regimen due to a lack of suitable guidance [4]. Patient’s own individual perceptions towards the relative barriers and benefits of physical activity and exercise may also contribute to their (non-)participation. Indeed, the perceived benefits and barriers towards a behaviour are important concepts of the health promotion model [5] and may influence physical activity behaviour [6].

Whilst the need for promoting physical activity and exercise participation in dialysis patients is widely recognized in international [7] and national guidelines [8], understanding individual-level barriers and benefits may help increase participation. As the two different treatment modalities themselves may affect patient preferences for the type and location of physical activity [6], it is important to understand any differences to better inform guidance in each modality. Identifying the barriers and benefits to exercise participation is an increasing area of research interest, however studies often focus on a single modality [9, 10] and/or have a small sample size [10, 11]. The association between perceived barriers and physical activity participation has only been explored in HD [12, 13]. Perceived benefits and physical activity behaviour have not yet been explored. This study aimed to (1) identify the barriers and benefits of exercise perceived by individuals on HD and PD and (2) to determine which have the greatest influence on whether individuals are physically active or not.

## Materials and methods

This is a secondary analysis of data from a cross-sectional observational multicentre study (ISRCTN87066351) [3]. Data were gathered between July 2012 and October 2018 from 17 sites across England. Ethical approval was granted by the East Midlands-Leicester South Research Ethics Committee and Health Research Authority (12/EM/0184). All participants provided written informed consent. The study was undertaken in accordance with the Declaration of Helsinki.

### Participants

Adults (aged ≥ 18 years) receiving prevalent (> 3 months) dialysis treatment (HD or PD) were eligible for inclusion. Patients were recruited from the waiting areas of hospital clinic appointments or the dialysis treatment unit. Participants were provided with a survey pack, which included the outcomes below.

### Outcome measures

#### Demographic and clinical characteristics

Clinical (time on dialysis where recorded, comorbidities) and demographic (sex, age, smoking status and ethnicity) data were taken from a composite of medical records and self-reported responses.

#### Exercise benefits and barriers

Individuals’ perceived benefits and barriers to exercise were evaluated using the 24-item ‘Dialysis Patient-perceived Exercise Benefits and Barriers Scale (DPEBBS)’ [14]. The DPEBBS is a dialysis-modified version of the ‘Exercise Benefits and Barriers Scale’ [5] which measures the perceived exercise benefits and barriers among the general population. Patients rate answers on a 4-point Likert scale from ‘1’ (‘strongly disagree’) to ‘4’ (‘strongly agree’). Barrier items are reverse coded. For this study, Q1 (‘Exercise helped reduce my medical costs’) was removed as it is not relevant for a UK population. For the purpose of this manuscript, each question of the DPEBBS has been abridged to its key point for clarity and to improve readability. Full un-abridged statements can be found in Supplementary material 1.

#### Physical activity status

Physical activity status was evaluated using the self-reported ‘General Practice Physical Activity Questionnaire’ (GPPAQ) from which patients were defined as either ‘active’, ‘moderately active’, ‘moderately inactive’ and ‘inactive’ [15, 16]. ‘Active’ corresponds as meeting current UK physical activity guidelines. The time spent performing different activities (e.g., housework, gardening, exercise) was recorded.

### Statistical analysis

Descriptive and frequency statistics were used to describe patient characteristics. Dichotomous and categorical variables are presented as percentages and continuous variables as mean [standard deviation (SD)] or median [interquartile range (IQR)] for non-normally distributed data. Participants without completed survey packs were excluded from the analysis. The number of missing data can be found in Supplementary material 2. Statistical analysis was performed using IBM SPSS 26 (USA). Statistical significance was accepted as a P < 0.05.

Each barrier and benefit was classed as a binary variable (i.e. yes and no). For patients to have an observed barrier or benefit, they must have scored ‘agree’ (3) or ‘strongly agree’ (4). Frequency analysis and Chi-squared tests were conducted to compare barriers and benefits perceived between modalities. Binominal logistic regression was performed to determine which perceived barriers and benefits had the biggest impact on whether a patient was physically inactive, to determine the impact of age (controlling for total number of comorbidities) on perceived barriers and benefits and the likelihood of being physically inactive, and to determine which demographic and clinical characteristics influenced the barriers and benefits reported. Data on physical activity levels has previously been reported [3]; however, it is repeated here to provide context for the rest of the analysis.

## Results

### Summary of participant characteristics

A total of 1339 dialysis patients were recruited (1155 HD and 184 PD). Of these, 1022 HD patients and 124 PD patients completed the DPEBBS. Basic characteristics between those excluded and those included did not differ (Supplementary material 3). Participant characteristics stratified by dialysis modality are displayed in Table 1. In summary, the mean age of participants was 62.9 (SD 15.4). Males represented 64% (736/1146) of the sample and 37% (413/1131) were from a non-White background. HD participants spent an average of 554.2 (SD 315.2) minutes on dialysis per week.

**Table 1:** Participant characteristics

Variable	HD (n = 1022)	PD (n = 124)
Age, years	63.1 (± 15.3)	62.1 (± 15.2)
Sex, n (%) male	654 (64%)	82 (66%)
Ethnicity		
White, n (%)	621 (62%)	97 (80%)
South Asian, n (%)	140 (14%)	14 (11%)
Asian other, n (%)	35 (3%)	2 (2%)
Black, n (%)	191 (19%)	9 (7%)
Other, n (%)	22 (2%)	–
Albumin, g/L	37.4 (± 5.7)	33.4 (± 7.5)
Haemoglobin, g/L	11.1 (± 1.4)	11.0 (± 1.6)
Body mass index, kg/m^2^	27.0 (± 7.7)	27.1 (± 6.4)
No. of comorbidities		
Mean	1.1 (± 1.1)	1.3 (± 1.1)
Median, IQR	1.0 (2.0)	1.0 (2.0)
Dialysis per week, min	554.2 (315.2)	–

### Current physical activity levels

The prevalence of insufficient physical activity was high, with 89% (909/1022) of HD and 86% (106/124) of PD patients not meeting physical activity levels recommended in current guidelines [17].

### Barriers and benefits to exercise

**Fig. 1:**
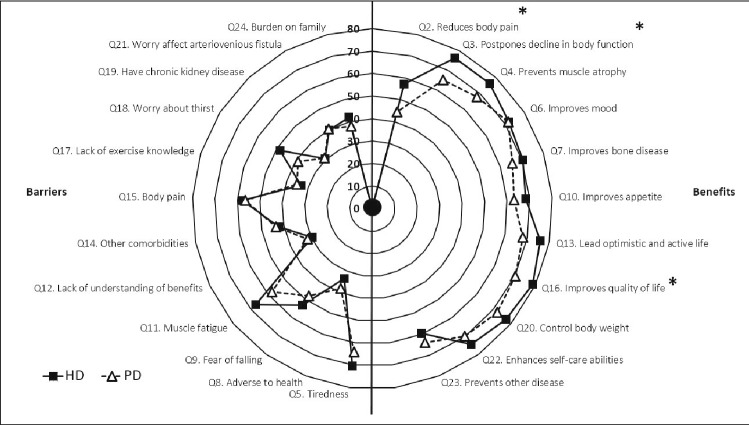
Radar plot showing the frequency of barriers and benefits to exercise reported by haemodialysis and peritoneal dialysis patients. Data presented as the prevalence (%) of patients in each group recognising each barrier or benefit (defined as agreeing or strongly agreeing to each variable). *HD* haemodialysis, *PD* peritoneal dialysis, *QoL* quality of life. *Significant (P < 0.050) difference between HD and PD groups. Data used to construct figure can be found in Supplementary material 5

The proportions of HD and PD patients who reported each barrier and benefit to exercise is displayed in Fig. 1. The most reported perceived benefit of exercise in HD patients was ‘improves quality of life (QoL)’ (reported by 79%). The most reported benefits in PD patients were ‘control body weight’ and ‘improves mood’ (both 72%). The most frequently reported barrier in both HD (70%) and PD (64%) patients was ‘tiredness’.

### Impact of perceived benefits and barriers on physical activity levels

Table 2 shows the differences in perceived exercise benefits and barriers stratified by physical activity level. Overall, a greater proportion of ‘active’ HD patients reported benefits of exercise than ‘inactive’ patients. Significant differences in the frequency of benefits was seen in 7 out of the 11 benefits. Conversely, a greater proportion of ‘inactive’ patients reported barriers to exercise than the ‘active’ group. Significant differences were seen in 9 out of the 12 barriers. No differences were seen between ‘active’ and ‘inactive’ PD patients.

**Table 2:** Differences in dialysis patient-perceived exercise benefits and barriers stratified by physical activity levels

Question	HD			PD		
	Active n = 113	Inactive N = 909	P	Active n = 18	Inactive n = 106	P
Benefits						
Q2. Reduces body pain	**77/107 (72%)**	464/841 (55%)	**0.001**	5/14 (36%)	42/92 (46%)	0.486
Q3. Postpones decline in body function	**97/109 (89%)**	628/841 (75%)	**0.001**	13/18 (72%)	62/97 (64%)	0.497
Q4. Prevents muscle atrophy	88/108 (81%)	630/835 (75%)	0.166	13/16 (81%)	61/93 (66%)	0.215
Q6. Improves mood	**93/ 109 (85%)**	595/849 (70%)	**0.001**	15/18 (83%)	68/98 (69%)	0.228
Q7. Improves bone disease	81/107 (76%)	556/799 (70%)	0.194	15/18 (83%)	53/86 (62%)	0.078
Q10. Improves appetite	82/109 (75%)	568/841 (68%)	0.104	12/18 (67%)	60/96 (63%)	0.737
Q13. Lead optimistic and active life	**98/109 (90%)**	623/839 (72%)	**< 0.001**	14/18 (78%)	63/95 (66%)	0.339
Q16. Improves quality of life	**100/110 (91%)**	651/841 (77%)	**0.001**	14/18 (78%)	66/96 (69%)	0.442
Q20. Control body weight	**92/108 (85%)**	640/841 (76%)	**0.034**	14/17 (82%)	66/94 (70%)	0.305
Q22. Enhances self-care abilities	**95/107 (89%)**	606/832 (73%)	**< 0.001**	12/17 (71%)	65/93 (70%)	0.954
Q23. Prevents other disease	67/107 (63%)	493/835 (59%)	0.478	10/17 (59%)	59/91 (65%)	0.636
Barriers						
Q5. Tiredness	64/109 (59%)	**611/851 (72%)**	**0.005**	12/17 (71%)	61 97 (63%)	0.542
Q8. Adverse to health	32/108 (30%)	282/830 (34%)	0.368	9/18 (50%)	34/95 (36%)	0.255
Q9. Fear of falling	39/107 (36%)	**470/855 (55%)**	**< 0.001**	8/18 (44%)	46/95 (48%)	0.757
Q11. Muscle fatigue	60/107 (56%)	**564/825 (68%)**	**0.011**	10/18 (56%)	52/89 (58%)	0.822
Q12. Lack of understanding of benefits	21/108 (19%)	**260/842 (31%)**	**0.014**	4/18 (22%)	32/95 (34%)	0.339
Q14. Other comorbidities	21/108 (19%)	**382/849 (45%)**	**< 0.001**	4/17 (24%)	45/96 (47%)	0.073
Q15. Body pain	47/106 (44%)	**502/842 (60%)**	**0.003**	7/17 (41%)	57/96 (59%)	0.163
Q17. Lack of exercise knowledge	23/107 (21%)	**291/838 (35%)**	**0.006**	6/17 (35%)	33/94 (35%)	0.988
Q18. Worry about thirst	53/107 (50%)	405/840 (48%)	0.797	7/17 (41%)	35/91 (38%)	0.833
Q19. Have chronic kidney disease	21/107 (20%)	**267/837 (32%)**	**0.009**	5/17 (29%)	29/93 (31%)	0.884
Q21. Worry affect arteriovenious fistula	44/106 (42%)	309/782 (40%)	0.694	2/7 (29%)	25/60 (42%)	0.504
Q24. Burden on family	22/108 (20%)	**372/831 (45%)**	**< 0.001**	5/17 (29%)	36/91 (40%)	0.429

Bold significant values are *P* < 0.050

*Note.* Differences between groups assessed using Chi-square test (χ^2^). Significance recognised as P < 0.050

*HD* haemodialysis, *PD* peritoneal dialysis

**Fig. 2:**
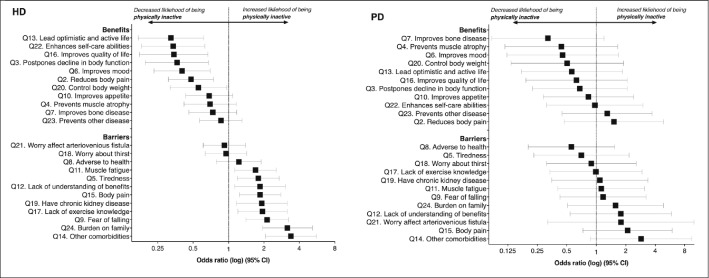
The association between barriers and benefits to exercise and the likelihood of being physically inactive in HD and PD patients. OR Odds ratio: an OR above 1 denotes an increased likelihood of being physically inactive; an OR of less than 1 denotes a decreased likelihood of being inactive. Values arranged in ascending order for each group. *HD* haemodialysis, *PD* peritoneal dialysis. Data used to construct figure can be found in Supplementary material 6

Figure 2 (data in Supplementary material 6) shows the association between barriers and benefits and the likelihood of being physically inactive in HD and PD patients. HD patients reporting ‘other comorbidities’ as a barrier were over three times more likely to be inactive (OR 3.389, P < 0.001). Individuals reporting exercise was a ‘burden on family’ were 3 times more likely to be inactive (OR 3.168, P < 0.001), whilst those who perceived ‘fear of falling’ as a barrier were 2 times more likely to be inactive (OR 2.129, P < 0.001). Those reporting ‘lack of exercise knowledge’ were twice as likely to be inactive (OR 1.943, P = 0.007). HD patients perceiving that exercise can ‘lead optimistic and active life’ (OR 0.324, P = 0.001) and that it can ‘enhance self-care abilities’ (OR 0.339, P = 0.001) were less likely to be inactive (i.e. more likely to be physically active). In PD, there were no differences in perceived benefits or barriers between those active and inactive. However, ‘other comorbidities’ were reported as a barrier in approximately double the number of inactive patients (active: 24% vs. inactive: 47%, P = 0.073). Perceived benefits and barriers had no impact on the likelihood of being active.

**Fig. 3:**
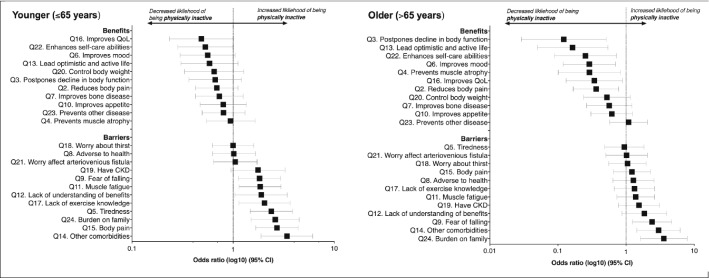
The association between barriers and benefits to exercise and the likelihood of being physically inactive in younger and older patients. *OR* odds ratio: an OR above 1 denotes an increased likelihood of being physically inactive; an OR of less than 1 denotes a decreased likelihood of being inactive. Values arranged in ascending order for each group. Data used to construct figure can be found in Supplementary material 7

Figure 3 (data in Supplementary material 7) shows the association between barriers and benefits and the likelihood of being physically inactive in older and younger patients (based on median age of sample). Younger patients reporting ‘tiredness’ (OR 2.375, P < 0.001), ‘body pain’ (OR 2.703, P < 0.001), ‘lack of exercise knowledge’ (OR 2.034, P = 0.016) were over 2 times more likely to be physically inactive. These barriers did not affect physical activity in older patients. Younger individuals who perceived ‘fear of falling’ (OR 1.820, P = 0.014), ‘muscle fatigue’ (OR 1.843, P = 0.012), and ‘lack of understanding the benefits’ (OR 1.891, P = 0.035) were more likely to be inactive. Older patients reporting ‘reduces body pain’ (OR 0.362, P = 0.010), ‘postpones decline in body function’ (OR 0.122, P = 0.004), ‘prevents muscle atrophy’ (OR 0.289, P = 0.021), ‘improves mood’ (OR 0.070, P = 0.006), ‘lead an optimistic and active life’ (OR 0.164, P = 0.003), and ‘improves QoL’ (OR 0.342 m P = 0.028) were less likely to be inactive (i.e. more likely to be physically active). These benefits had no impact on the likelihood of being active in younger patients.

### Factors predicting barriers and benefits reported

HD patients who reported ‘lack of understanding benefits’ (OR 1.013, P = 0.017), ‘other comorbidities’ (OR 1.018, P < 0.001), and ‘having CKD’ (OR 1.017, P = 0.002) as barriers to exercise were more likely to be older. Individuals reporting ‘worry about thirst’ (OR 0.986, P = 0.005) and ‘worry affect arteriovenious fistula’ (OR 0.985, P = 0.003) as barriers to exercise were more likely to be younger. Females on HD were more likely to report ‘fear of falling’ (OR 1.392, P = 0.036) and ‘burden on family’ (OR 1.450, P = 0.021) as barriers. Those reporting ‘muscle fatigue’ as a barrier were more likely to have lower haemoglobin levels (OR 0.841, P = 0.003). HD patients reporting ‘improves mood’ (OR 0.982, P = 0.002), ‘improves appetite’ (OR 0.986, P = 0.011), ‘lead optimistic and active life’ (OR 0.984, P = 0.006), ‘improves QoL’ (OR 0.976, P = 0.001), and ‘controls body weight’ (OR 0.720, P = 0.002) as benefits to exercise were more likely to be younger. Individuals reporting ‘prevents other diseases’ (OR 0.637, P = 0.005) as a benefit were more likely to be male. (Supplementary material 8).

## Discussion

People on dialysis are extremely inactive despite the myriad of potential benefits available to this population [3]. Identifying the barriers and benefits to exercise perceived by patients on different modalities of dialysis may help develop better interventions to address physical inactivity. Our findings show that in both HD and PD groups the most reported barrier to exercise is tiredness, closely followed by muscle fatigue and body pain. The most frequently reported benefit to exercise by HD patients was ‘improves QoL’ whereas for PD patients this was to ‘control body weight’. Of the barriers reported, ‘other comorbidities’ and ‘burden on family’ had the largest effect on physical activity levels in those on HD. The perceived benefits and barriers between active and inactive PD patients did not differ, nor did they impact physical activity levels.

The most frequent barriers to exercise reported by both groups relate to symptoms commonly reported by CKD patients such as tiredness and pain [18]. Previous research exploring barriers to exercise identified fatigue, often viewed as concomitant with tiredness by patients [19], as a commonly reported and major barrier to exercise for patients receiving HD [11, 20]. Fatigue, defined by people on dialysis as feeling tired and without energy for most of the time [21], has been identified as an independent predictor for decreased physical activity in PD and HD populations [22], with those reporting higher levels of fatigue engaging in the least number of activities [23]. Lack of energy due to the disease or dialysis treatment may result in low levels of physical activity which in turn may perpetuate fatigue further [20]. Fatigue is one of the most prevalent and distressing symptoms contributing to symptom burden, however, it is often overlooked by clinicians and underreported by patients and therefore warrants routine assessment [24]. Improvements in energy levels is a recognised benefit of exercise and thus should be promoted to patients, particularly as these individuals value improvements in fatigue so highly [23]. Given that fatigue has been identified as a core outcome and symptom of critical importance for research [25], interventions to mitigate its effects, including the promotion of physical activity, should be encouraged.

Body pain, a symptom affecting over half of individuals treated with dialysis [26], was identified as a key barrier to physical activity participation by HD and PD patients. Pain is underreported by patients due to beliefs that they would be ignored or dismissed by clinicians [27]; as a result, pain is often unrecognised and inadequately addressed in clinical practice [28, 29]. Despite pain being identified as a key barrier, the benefit of exercise ‘reduces body pain’ was the least frequently reported perceived benefit by both groups. Such discrepancy suggests this potential benefit of exercise is poorly understood. A third of patients reported a ‘lack of understanding of benefits’ and ‘lack of exercise knowledge’ as barriers to exercise, and those receiving HD reporting these barriers were twice as likely to be inactive. Given that education regarding exercise is not part of routine practice in dialysis care [30], it is likely that patients are unsure about appropriate guidance. Patients rely heavily on healthcare providers for support, encouragement and guidance regarding exercise, however patients have reported receiving limited exercise counselling [20, 31]. As such, there is a need to increase healthcare professionals’ engagement and involvement in the prescription of exercise, and the promotion of the benefits of physical activity is likely to have positive effects on activity engagement.

Perceived ‘other comorbidities’ had the largest effect on physical activity levels; those perceiving it as a barrier were over 3 times more likely to be inactive. This finding is similar to others [12, 13]. Comorbidities may impact physical activity through increased pain, fatigue, and decreased physical function [32]. It could be presumed that these patients may likely have more comorbidities themselves; however, we found no difference in the number of comorbidities between those who viewed ‘other comorbidities’ as a barrier and those whom did not. However, older HD patients were more likely to report having CKD and other comorbidities as barriers. Poor physical condition as a result of both co-morbid conditions and CKD-related symptoms (fatigue; joint pain and shortness of breath) [33] and advancing age may cause concerns about CKD aggravation as a result of over-doing exercise [34]. Interventions to increase physical activity should address concerns of ‘other comorbidities’ as a barrier and promote physical activity as a means to manage other health conditions. As ‘prevents other diseases’ was a poorly reported benefit to exercise, it may be that the broader benefits of physical activity are not well understood. Thus, when promoting physical activity, there is a need to focus on educating patients about the broader benefits of exercise, such as improvements in tolerance to dialysis and management of health risk factors, other comorbidities and symptoms [35].

The perceived ‘burden on family’ also had a large effect on physical activity status, although it is unclear whether this is viewed as a need for social support or for safety. Nonetheless, family support is highly valued by patients and having someone to exercise with is a motivator to exercise participation [36]. Females were more likely to report ‘burden on family’ as a barrier to exercise. Given that the number of children can influence the number of barriers perceived by women [37], providing exercise information and plans which include family members may be a way of promoting exercise [38]. In addition, encouraging family members to support physical activity may confer positive effects on participation. Patients may also prefer company whilst exercising, particularly outdoors, for safety reasons. Concerns for safety, including risk of falling, have previously been reported and are associated with lower physical activity levels [11]. In our study 19% more inactive people reported ‘fear of falling’ as a barrier compared to active patients. Fear of falling can lead to limitation of activities and leaving the home less frequently [39], which, in turn, can lead to decreased strength, agility and balance resulting in loss of independence, functional decline, and increased falling itself [40]. Reducing the risks of engaging in physical activity can be easily addressed (e.g. with balance exercises) and concerns about safety should not be absolute contraindications to physical activity [11].

Muscle fatigue is considered important in dialysis patients’ overall physical capacity. We found those reporting muscle fatigue as a barrier to exercise were more likely to have lower levels of haemoglobin—a protein with an important oxygen-carrying role. It is unsurprising that low haemoglobin levels are associated with decreased muscle and physical function [33]. Although anaemia may limit exercise capacity and physical functioning, levels of haemoglobin have been shown to have no association with physical activity levels [41]. Aside from adequate management of anaemia, resistance training in combination with aerobic training results in significant improvements in muscle health [34] and can lead to reductions in perceived weakness and the number of ‘loss of muscular strength/power’ symptoms reported [42].

Improvements in QoL, mood, and self-care abilities were the most commonly reported benefits of exercise in our study. These results are consistent with other studies which have explored the perceived barriers and benefits of exercise in HD [9] and PD patients [10]. Unsurprisingly, active HD patients overall reported more benefits and fewer barriers to exercise than those who were inactive. The most commonly reported benefits to exercise for ‘active’ patients were largely psychologically based (e.g., improves mood), whereas the most frequently reported for ‘inactive’ patients were physiological (e.g., controls body weight). Exercise as a mechanism to improve QoL as opposed to reducing comorbidities (e.g. heart disease) and hospitalisation is perhaps of greater importance to patients [43]. Self-awareness of the benefits of exercise has been identified as a key motivator for exercise participation and adherence [20], alongside experiencing positive benefits from exercise and achieving health goals [11, 20]. Individuals with a desire to maintain or improve functional ability are more likely to increase their physical activity levels and achieve exercise goals [11]. Given that motivation is important in behaviour change and is known to be a strong facilitator of self-directed exercise [36], interventions to increase exercise participation should target patient autonomy and self-efficacy [36].

Whilst the perceived benefits and barriers between active and inactive PD patients did not differ, there may be other barriers impacting their physical activity levels. Perceived barriers, such as catheter healing, dressing and water, intraabdominal pressure, and hernias, can result in PD patients being discouraged from participating in exercise programmes [4]. In addition, PD patients may not be encouraged to exercise due to a lack of uncertainty about the most appropriate exercise programme for this population [4]. Most PD patients dialyse at home and are less accessible to clinical staff, thus exercise programme delivery is more challenging in this group compared to HD patients [44], who spend several hours each week receiving treatment during which exercise programmes can be tested that potentially enhance participation rates that may not be the same for PD patients who are at home [45].

Perceived barriers and benefits to physical activity and exercise are considered to change over an individual’s life course [46, 47], it is unsurprising that differences were observed in the perceived barriers and benefits to exercise and the effects on physical activity status in the younger and older patients. Potential reasons for this may be that older individuals are more motivated by the benefits of exercise as they consider physical activity to have the potential to preserve and improve their physical and mental health [46, 47]. Exercise interventions should be age-appropriate and focus on promoting the specific benefits and addressing barriers perceived by different age groups.

The study has several limitations. Whilst smaller than the HD group, the number of PD patients included is greater than those seen in other studies [10, 11] and represents the approximate proportion (~ 11%) of global PD use compared to HD [48]. The cross-sectional design of this study prevents the analysis of behaviours over time and does not allow causation to be determined. However, the findings of this study can be used to guide interventions which promote the benefits and address the barriers perceived. Unlike others, who developed their own questionnaires to investigate barriers and physical activity participation [12, 13], we used a recognised questionnaire to assess the barriers and benefits of exercise. We acknowledge that the DPEBBS assessed perceptions around exercise, which is different from the broader concept of physical activity. Nonetheless, all of the barriers and benefits in the DPEBBS are relevant to physical activity, and not necessarily specific to exercise. Whilst further studies are needed to better characterise the benefits (and risks) of exercise, targeted interventions addressing the barriers and promoting the benefits identified here may facilitate greater exercise participation. Future qualitative studies are required to further explore approaches required for specific stages of change and to understand patients’ wants and needs for an exercise-based behavioural change intervention.

## Conclusion

Although it appears most patients are aware of the benefits of exercise, people on dialysis are extremely inactive. Individuals perceive several barriers which may prevent them from engaging in physical activity. Our findings highlight the need for interventions targeting and addressing barriers to exercise that patients perceive to be the most important. In addition, broader changes must be implemented to increase patients’ willingness to change and modify their current exercise behaviours and to maintain new exercise behaviours. The approach when counselling patients about how to engage in and increase their physical activity levels needs to be tailored to the individual and to dialysis modality in order to improve exercise self-efficacy and exercise-related behaviours.

## Supplementary Information

Below is the link to the electronic supplementary material.

Supplementary file1 (DOCX 49 kb)

## Data Availability

Data available in Supplementary material.

## References

[1] Thangarasa T, Imtiaz R, Hiremath S, Zimmerman D (2018). Physical activity in patients treated with peritoneal dialysis: a systematic review and meta-analysis. Can J Kidney Health Dis.

[2] Huang M, Lv A, Wang J, Xu N, Ma G, Zhai Z, Zhang B, Gao J, Ni C (2019). Exercise training and outcomes in hemodialysis patients: systematic review and meta-analysis. Am J Nephrol.

[3] Wilkinson TJ, Clarke AL, Nixon DGD, Hull KL, Song Y, Burton JO, Yates T, Smith AC (2019). Prevalence and correlates of physical activity across kidney disease stages: an observational multicentre study. Nephrol Dial Transplant.

[4] Thangarasa T, Imtiaz R, Hiremath S, Zimmerman D (2017). Physical activity in patients treated with peritoneal dialysis: a protocol for a systematic review. Can J Kidney Health Dis.

[5] Sechrist KR, Walker SN, Pender NJ (1987). Development and psychometric evaluation of the exercise benefits/barriers scale. Res Nurs Health.

[6] Brown SA (2005). Measuring perceived benefits and perceived barriers for physical activity. Am J Health Behav.

[7] Foundation NK, Workgroup KD (2005). K/DOQI clinical practice guidelines for cardiovascular disease in dialysis patients. Am J Kidney Dis.

[8] Ashby D, Borman N, Burton J, Corbett R, Davenport A, Farrington K, Flowers K, Fotheringham J, Andrea Fox RN, Franklin G, Gardiner C, Martin Gerrish RN, Greenwood S, Hothi D, Khares A, Koufaki P, Levy J, Lindley E, Macdonald J, Mafrici B, Mooney A, Tattersall J, Tyerman K, Villar E, Wilkie M (2019). Renal association clinical practice guideline on haemodialysis. BMC Nephrol.

[9] Jayaseelan G, Bennett PN, Bradshaw W, Wang W, Rawson H (2018). Exercise benefits and barriers: the perceptions of people receiving hemodialysis. Nephrol Nurs J.

[10] Zeng J, Bennett PN, Hill K, Borlace M, Xu Q (2020). The exercise perceptions of people treated with peritoneal dialysis. J Ren Care.

[11] Sheshadri A, Kittiskulnam P, Delgado C, Sudore R, Lai JC, Johansen KL (2020). Association of motivations and barriers with participation and performance in a pedometer-based intervention. Nephrol Dial Transplant.

[12] Delgado C, Johansen KL (2012). Barriers to exercise participation among dialysis patients. Nephrol Dial Transplant.

[13] Fiaccadori E, Sabatino A, Schito F, Angella F, Malagoli M, Tucci M, Cupisti A, Capitanini A, Regolisti G (2014). Barriers to physical activity in chronic hemodialysis patients: a single-center pilot study in an Italian dialysis facility. Kidney Blood Press Res.

[14] Zheng J, You LM, Lou TQ, Chen NC, Lai DY, Liang YY, Li YN, Gu YM, Lv SF, Zhai CQ (2010). Development and psychometric evaluation of the dialysis patient-perceived Exercise Benefits and Barriers Scale. Int J Nurs Stud.

[15] Heron N, Tully MA, McKinley MC, Cupples ME (2014). Physical activity assessment in practice: a mixed methods study of GPPAQ use in primary care. BMC Fam Pract.

[16] Ahmad S, Harris T, Limb E, Kerry S, Victor C, Ekelund U, Iliffe S, Whincup P, Beighton C, Ussher M, Cook DG (2015). Evaluation of reliability and validity of the General Practice Physical Activity Questionnaire (GPPAQ) in 60–74 year old primary care patients. BMC Fam Pract.

[17] Department of Health & Social Care (DHSC) (2019) UK Chief Medical Officers’ Physical Activity Guidelines. https://assets.publishing.service.gov.uk/government/uploads/system/uploads/attachment_data/file/832868/ukchief-medical-officers-physical-activity-guidelines.pdf

[18] Brown SA, Tyrer FC, Clarke AL, Lloyd-Davies LH, Stein AG, Tarrant C, Burton JO, Smith AC (2017). Symptom burden in patients with chronic kidney disease not requiring renal replacement therapy. Clin Kidney J.

[19] Picariello F, Moss-Morris R, Macdougall IC, Chilcot J (2018). 'It's when you're not doing too much you feel tired': a qualitative exploration of fatigue in end-stage kidney disease. Br J Health Psychol.

[20] Jhamb M, McNulty ML, Ingalsbe G, Childers JW, Schell J, Conroy MB, Forman DE, Hergenroeder A, Dew MA (2016). Knowledge, barriers and facilitators of exercise in dialysis patients: a qualitative study of patients, staff and nephrologists. BMC Nephrol.

[21] Urquhart-Secord R, Craig JC, Hemmelgarn B, Tam-Tham H, Manns B, Howell M, Polkinghorne KR, Kerr PG, Harris DC, Thompson S, Schick-Makaroff K, Wheeler DC, van Biesen W, Winkelmayer WC, Johnson DW, Howard K, Evangelidis N, Tong A (2016). Patient and caregiver priorities for outcomes in hemodialysis: an International Nominal Group Technique Study. Am J Kidney Dis.

[22] Painter PL, Agarwal A, Drummond M (2017). Physical function and physical activity in peritoneal dialysis patients. Perit Dial Int.

[23] Ramkumar N, Beddhu S, Eggers P, Pappas LM, Cheung AK (2005). Patient preferences for in-center intense hemodialysis. Hemodial Int.

[24] Almutary H, Bonner A, Douglas C (2016). Which patients with chronic kidney disease have the greatest symptom burden? A comparative study of advanced CKD stage and dialysis modality. J Ren Care.

[25] Ju A, Unruh M, Davison S, Dapueto J, Dew MA, Fluck R, Germain M, Jassal SV, Obrador G, O’Donoghue D, Josephson MA, Craig JC, Viecelli A, O’Lone E, Hanson CS, Manns B, Sautenet B, Howell M, Reddy B, Wilkie C, Rutherford C, Tong A, Levin A, Narva A, Wang A, Ralph A, Moffat AM, Bell B, Hemmelgarn B, Schiller B, Hawley C, Perry C, Wanner C, Cukor D, Perez D, Cella D, Harris D, Johnson D, Roer D, Van Wyck D, Wheeler D, Deyhle D, Gill D, Schatell D, Bavlovlenkov E, Weinhandl E, Caskey F, Tentori F, Sakkas G, Saver H, Wells H, Wadee J, Akbar J, Carter J, Flythe J, Shen J, Kusek J, Gill J, Beverly J, Pinter J, Johansen K, Meyer K, Lirtzman L, Wagner-Weiner L, Costabile L, Jhamb M, Tonelli M, Ruospo M, Howell M, Bossola M, Thomas M, Mendez N, Powe N, Gedney N, Rouse N, Kaden P, Kerr P, Tugwell P, Taylor Q, Sand R, Pecoits-Filho R, Crowe S, Gill S, Jowsey-Gregoire S, Fadem S, McDonald S, Weisbord S, Palmer S, Hedayati SS, Harris T, Hiemstra TF, Muhammed U, McNorton V, Sikirica V, Jha V, Herrington W, Van Biesen W, Winkelmayer W, Butt Z (2018). Establishing a core outcome measure for fatigue in patients on hemodialysis: a standardized outcomes in nephrology-hemodialysis (SONG-HD) Consensus Workshop Report. Am J Kidney Dis.

[26] Santoro D, Satta E, Messina S, Costantino G, Savica V, Bellinghieri G (2013). Pain in end-stage renal disease: a frequent and neglected clinical problem. Clin Nephrol.

[27] Zhang K, Hannan E, Scholes-Robertson N, Baumgart A, Guha C, Kerklaan J, Hanson CS, Craig JC, Davison SN, Hecking M, Tong A (2020). Patients' perspectives of pain in dialysis: systematic review and thematic synthesis of qualitative studies. Pain.

[28] Davison SN, Koncicki H, Brennan F (2014). Pain in chronic kidney disease: a scoping review. Semin Dial.

[29] Brkovic T, Burilovic E, Puljak L (2016). Prevalence and severity of pain in adult end-stage renal disease patients on chronic intermittent hemodialysis: a systematic review. Patient Prefer Adherence.

[30] Bennett PN, Capdarest-Arest N, Parker K (2017). The physical deterioration of dialysis patients-Ignored, ill-reported, and ill-treated. Semin Dial.

[31] Thompson S, Tonelli M, Klarenbach S, Molzahn A (2016). A qualitative study to explore patient and staff perceptions of intradialytic exercise. Clin J Am Soc Nephrol.

[32] Steeves JA, Shiroma EJ, Conger SA, Van Domelen D, Harris TB (2019). Physical activity patterns and multimorbidity burden of older adults with different levels of functional status: NHANES 2003–2006. Disabil Health J.

[33] Penninx BW, Pahor M, Cesari M, Corsi AM, Woodman RC, Bandinelli S, Guralnik JM, Ferrucci L (2004). Anemia is associated with disability and decreased physical performance and muscle strength in the elderly. J Am Geriatr Soc.

[34] Watson EL, Gould DW, Wilkinson TJ, Xenophontos S, Clarke AL, Vogt BP, Viana JL, Smith AC (2018). Twelve-week combined resistance and aerobic training confers greater benefits than aerobic training alone in nondialysis CKD. Am J Physiol Ren Physiol.

[35] Barcellos FC, Santos IS, Umpierre D, Bohlke M, Hallal PC (2015). Effects of exercise in the whole spectrum of chronic kidney disease: a systematic review. Clin Kidney J.

[36] Clarke AL, Young HM, Hull KL, Hudson N, Burton JO, Smith AC (2015). Motivations and barriers to exercise in chronic kidney disease: a qualitative study. Nephrol Dial Transplant.

[37] Hickey ME, Mason SE (2017). Age and gender differences in particpation rates, motivators for, and barriers to exercise. Mod Psychol Stud.

[38] Clarke AL, Jhamb M, Bennett PN (2019). Barriers and facilitators for engagement and implementation of exercise in end-stage kidney disease: future theory-based interventions using the Behavior Change Wheel. Semin Dial.

[39] van Loon IN, Joosten H, Iyasere O, Johansson L, Hamaker ME, Brown EA (2019). The prevalence and impact of falls in elderly dialysis patients: frail elderly Patient Outcomes on Dialysis (FEPOD) study. Arch Gerontol Geriatr.

[40] Murphy SL, Williams CS, Gill TM (2002). Characteristics associated with fear of falling and activity restriction in community-living older persons. J Am Geriatr Soc.

[41] Johansen KL, Chertow GM, Kutner NG, Dalrymple LS, Grimes BA, Kaysen GA (2010). Low level of self-reported physical activity in ambulatory patients new to dialysis. Kidney Int.

[42] Wilkinson TJ, Watson EL, Gould DW, Xenophontos S, Clarke AL, Vogt BP, Viana JL, Smith AC (2018). Twelve weeks of supervised exercise improves self-reported symptom burden and fatigue in chronic kidney disease: a secondary analysis of the ‘ExTra CKD’ trial. Clin Kidney J.

[43] Moorman D, Suri R, Hiremath S, Jegatheswaran J, Kumar T, Bugeja A, Zimmerman D (2019). Benefits and barriers to and desired outcomes with exercise in patients with ESKD. Clin J Am Soc Nephrol.

[44] Isnard-Rouchon M, West M, Bennett PN (2019). Exercise and physical activity for people receiving peritoneal dialysis: why not?. Semin Dial.

[45] Ellam T, Wilkie M (2007). Peritoneal dialysis. Medicine.

[46] Moschny A, Platen P, Klaaßen-Mielke R, Trampisch U, Hinrichs T (2011). Barriers to physical activity in older adults in Germany: a cross-sectional study. Int J Behav Nutr Phys Act.

[47] Han BH, Sadarangani T, Wyatt LC, Zanowiak JM, Kwon SC, Trinh-Shevrin C, Lee L, Islam NS (2016). Correlates of physical activity among middle-aged and older Korean Americans at Risk for Diabetes. J Nurs Scholarsh.

[48] Jain AK, Blake P, Cordy P, Garg AX (2012). Global trends in rates of peritoneal dialysis. J Am Soc Nephrol.

