# Supplementary material for: Perceptions of exercise benefits and barriers: the influence on physical activity behaviour in individuals undergoing haemodialysis and peritoneal dialysis
Source: J Nephrol. 2021 Mar 26;34(6):1961–71. doi: 10.1007/s40620-021-01024-y (PMC8610943; doi:10.1007/s40620-021-01024-y)
Supplement: Supplementary file 1 — Supplementary file1 (DOCX 49 kb) [file 40620_2021_1024_MOESM1_ESM.docx]

**Supplementary Material 1. Dialysis patient-perceived Exercise Benefits and Barriers Scale questions and abridged key points**

| **Full question** | **Abridged key points** |
| --- | --- |
| 1. Exercise helps reduce my body pain | Reduces body pain |
| 1. Exercise can postpone a decline in body function | Postpones decline in body function |
| 1. Exercise helps prevent muscular atrophy | Prevents muscle atrophy |
| 1. Frequent tiredness impedes my exercise participation | Tiredness |
| 1. Exercise improves my mood | Improves mood |
| 1. Exercise improves bone diseases | Improves bone disease |
| 1. Exercise is adverse to health of dialysis patients | Adverse to health |
| 1. I worry about a fall during exercise | Fear of falling |
| 1. Exercise improves my appetite | Improves appetite |
| 1. Frequent lower-extremity muscle fatigue impedes my exercise participation | Muscle fatigue |
| 1. I lack an understanding of the benefits of exercise | Lack of understanding of benefits |
| 1. Exercise helps me lead an optimistic and active life | Lead optimistic and active life |
| 1. Exercise is not suitable for me since I have comorbidities | Other comorbidities |
| 1. Body pain impedes my exercise participation | Body pain |
| 1. Exercise improves my quality of life | Improves quality of life |
| 1. I lack an understanding of the knowledge how to carry out exercise | Lack of exercise knowledge |
| 1. I worry that exercise may make me thirsty | Worry about thirst |
| 1. Exercise is not suitable for me since I have kidney disease | Have chronic kidney disease |
| 1. Exercise can keep my body weight at a steady level | Control body weight |
| 1. I worry that exercise may affect arteriovenous fistula | Worry affect arteriovenious fistula |
| 1. Exercise helps enhance my self-care abilities | Enhances self-care abilities |
| 1. Exercise will keep me from having other diseases (e.g. cold) | Prevents other disease |
| 1. Outdoor exercise adds burden to my family since I need their company while I am out | Burden on family |

**Supplementary Material 2. Frequency of missing data**

| **Variable** | **HD** | **PD** |
| --- | --- | --- |
|  | **n=1022** | **n=124** |
| Gender | 5 (0.2%) | 1 (1%) |
| Age | 5 (3%) | 1 (1%) |
| Ethnicity | 13 (1%) | 2 (2%) |
| Smoking status | 18 (18%) | 0 (0%) |
| eGFR | 562 (55%) | 10 (8%) |
| Hb | 278 (27%) | 11 (9%) |
| Total no. comorbidities | 0 (0%) | 0 (0%) |
| Dialysis per week, minutes | 36 (4%) | - |
| **DPPEBABS questions** | | |
| Q2. Reduces body pain | 74 (7%) | 18 (15%) |
| Q3. Postpones decline in body function | 72 (7%) | 9 (7%) |
| Q4. Prevents muscle atrophy | 79 (7%) | 15 (10%) |
| Q5. Tiredness | 62 (6%) | 10 (5%) |
| Q6. Improves mood | 64 (7%) | 8 (9%) |
| Q7. Improves bone disease | 116 (11%) | 20 (16%) |
| Q8. Adverse to health | 84 (8%) | 11 (9%) |
| Q9. Fear of falling | 60 (6%) | 11 (9%) |
| Q10. Improves appetite | 72 (7%) | 10 (8%) |
| Q11. Muscle fatigue | 90 (9%) | 17 (14%) |
| Q12. Lack of understanding of benefits | 72 (7%) | 11 (9%) |
| Q13. Lead optimistic and active life | 74 (7%) | 11 (9%) |
| Q14. Other comorbidities | 65 (6%) | 11 (9%) |
| Q15. Body pain | 74 (7%) | 11 (9%) |
| Q16. Improves quality of life | 71 (7%) | 10 (8%) |
| Q17. Lack of exercise knowledge | 77 (8%) | 13 (11%) |
| Q18. Worry about thirst | 75 (7%) | 16 (13%) |
| Q19. Have chronic kidney disease | 78 (8%) | 14 (11%) |
| Q20. Control body weight | 73 (7%) | 13 (11%) |
| Q21. Worry affect arteriovenious fistula | 134 (13%) | 57 (46%) |
| Q22. Enhances self-care abilities | 83 (8%) | 14 (11%) |
| Q23. Prevents other disease | 80 (8%) | 16 (13%) |
| Q24. Burden on family | 83 (8%) | 16 (13%) |
| *Note.*  Data displayed as missing n (%)  DPPEBABS = Dialysis patient-perceived Exercise Benefits and Barriers Scale | | |

**Supplementary Material 3. Characteristics of participants excluded from analysis**

| **Variable** | **HD (n=133)** | **PD (n=60)** |
| --- | --- | --- |
| Age, years | 63.6 (±15.9) | 62.0 (±16.7) |
| Sex, n (%) male | 76 (63%) | 22 (65%) |
| Ethnicity |  |  |
| *White, n (%)* | 74 (70%) | 24 (78%) |
| *South Asian, n (%)* | 12 (11%) | 2 (6%) |
| *Asian other, n (%)* | 4 (4%) | 1 (3%) |
| *Black, n (%)* | 14 (13%) | 3 (10%) |
| *Other, n (%)* | 2 (2%) | 1 (3%) |
| Body mass index, kg/m^2^ | 28.3 (±8.6) | 27.6 (±5.9) |
| No. of comorbidities |  |  |
| *Mean* | 1.1 (±1.3) | 1.1 (±0.9) |
| *Median, IQR* | 1.0 (1.0) | 1.0 (1.0) |
| Dialysis per week, minutes | 553.0 (305.8) | Not reported |
| *Note.*  Data presented as mean and standard deviation, unless otherwise stated  HD = Haemodialysis; PD = Peritoneal dialysis; eGFR = Estimated glomerular filtration rate | | |

**Supplementary Material 4. Differences in characteristics between active and inactive participants**

| **Variable** | **HD (n=1022)** | | **PD (n=124)** | |
| --- | --- | --- | --- | --- |
|  | **Active**  **(n=113)** | **Inactive (n=909)** | **Active**  **(n=18)** | **Inactive (n=106)** |
| Age, years | 57.0 (±15.6) | 63.8 (±15.1) | 55.7 (±16.6) | 63.2 (±14.8) |
| Sex, n (%) male | 83 (74%) | 571 (63%) | 12 (67%) | 70 (66%) |
| Ethnicity |  |  |  |  |
| *White, n (%)* | 58 | 563 | 16 | 81 |
| *South Asian, n (%)* | 23 | 117 | 1 | 13 |
| *Asian other, n (%)* | 7 | 28 | - | 2 |
| *Black, n (%)* | 21 | 170 | 18 |  |
| *Other, n (%)* | 4 | 18 | - | - |
| Albumin, g/L | 38.7 (±8.1) | 37.4 (±5.3) | 34.0 (±5.7) | 32.5 (±6.3) |
| Haemoglobin, g/L | 11.7 (±1.4) | 11.1 (±1.4) | 10.3 (±2.2) | 11.0 (±1.6) |
| Body mass index, kg/m^2^ | 27.2 (±5.1) | 26.9 (±7.7) | 25.0 (±5.5) | 26.9 (±6.8) |
| No. of comorbidities |  |  |  |  |
| *Mean* | 1.0 (±0.9) | 1.1 (±1.1) | 1.3 (±2.3) | 1.2 (±1.1) |
| *Median, IQR* | 1.0 (±2.0) | 1.0 (±2.0) | 1.0 (±2.0) | 1.0 (±2.0) |
| Dialysis per week, minutes | 608.5 (±303.4) | 552.3 (±305.0) | - | - |

**Supplementary Material 5.** **Barriers and benefits to exercise reported by haemodialysis and peritoneal dialysis patients (data used in Figure 1)**

| **Questions** | **HD** | | **PD** | | **P** |
| --- | --- | --- | --- | --- | --- |
|  | **n=1022** | **Rank** | **n=124** | **Rank** |  |
| **Benefits** | | | | | |
| Q2. Reduces body pain | 541 (57%) | 11 | 47 (44%) | 11 | **0.012** |
| Q3. Postpones decline in body function | 725 (76%) | 3= | 75 (65%) | 7= | **0.009** |
| Q4. Prevents muscle atrophy | 718 (76%) | 3= | 74 (68%) | 5= | 0.059 |
| Q6. Improves mood | 688 (72%) | 7 | **83 (72%)** | **1=** | 0.952 |
| Q7. Improves bone disease | 637 (70%) | 8 | 68 (65%) | 7= | 0.300 |
| Q10. Improves appetite | 650 (68%) | 9 | 72 (63%) | 10 | 0.256 |
| Q13. Lead optimistic and active life | 721 (76%) | 3= | 77 (68%) | 5= | 0.066 |
| Q16. Improves quality of life | **751 (79%)** | **1** | 80 (70%) | 3= | **0.032** |
| Q20. Control body weight | 732 (77%) | 2 | **80 (72%)** | **1=** | 0.233 |
| Q22. Enhances self-care abilities | 701 (75%) | 6 | 77 (70%) | 3= | 0.291 |
| Q23. Prevents other disease | 560 (59%) | 10 | 69 (64%) | 9 | 0.372 |
| **Barriers** | | | | | |
| Q5. Tiredness | **675 (70%)** | **1** | **73 (64%)** | **1** | 0.168 |
| Q8. Adverse to health | 314 (33%) | 9= | 43 (38%) | 8= | 0.332 |
| Q9. Fear of falling | 509 (53%) | 4 | 54 (48%) | 4 | 0.302 |
| Q11. Muscle fatigue | 624 (67%) | 2 | 62 (58%) | 2 | 0.062 |
| Q12. Lack of understanding of benefits | 281 (30%) | 9= | 36 (32%) | 11 | 0.617 |
| Q14. Other comorbidities | 403 (42%) | 6= | 49 (43%) | 5 | 0.799 |
| Q15. Body pain | 549 (58%) | 3 | 64 (57%) | 3 | 0.795 |
| Q17. Lack of exercise knowledge | 314 (33%) | 10 | 39 (35%) | 10 | 0.687 |
| Q18. Worry about thirst | 458 (48%) | 5 | 42 (39%) | 7 | 0.062 |
| Q19. Have chronic kidney disease | 288 (31%) | 11 | 34 (31%) | 12 | 0.931 |
| Q21. Worry affect arteriovenious fistula | 353 (40%) | 8 | 27 (40%) | 6 | 0.930 |
| Q24. Burden on family | 394 (42%) | 6= | 41 (38%) | 8= | 0.425 |
| Note. | | | | | |

**Supplementary Material 6. The association between barriers and benefits to exercise and the likelihood of being physically inactive in HD and PD patients (data used in Figure 2)**

| **Questions** | **HD (n=1022)** | | | | **PD (n=124)** | | | |
| --- | --- | --- | --- | --- | --- | --- | --- | --- |
|  | **OR** | **Lower CI (95%)** | **Upper CI (95%)** | **P** | **OR** | **Lower CI (95%)** | **Upper CI (95%)** | **P** |
| **Benefits** | | | | | | | | |
| Q2. Reduces body pain | 0.480 | 0.308 | 0.747 | **0.001** | 1.512 | 0.470 | 4.860 | 0.488 |
| Q3. Postpones decline in body function | 0.365 | 0.196 | 0.678 | **0.001** | 0.681 | 0.224 | 2.071 | 0.499 |
| Q4. Prevents muscle atrophy | 0.698 | 0.419 | 1.164 | 0.168 | 0.440 | 0.117 | 1.657 | 0.225 |
| Q6. Improves mood | 0.403 | 0.232 | 0.699 | **0.001** | 0.453 | 0.122 | 1.683 | 0.237 |
| Q7. Improves bone disease | 0.734 | 0.461 | 1.171 | 0.195 | 0.321 | 0.086 | 1.195 | 0.090 |
| Q10. Improves appetite | 0.685 | 0.433 | 1.083 | 0.106 | 0.833 | 0.288 | 2.414 | 0.737 |
| Q13. Lead optimistic and active life | 0.324 | 0.170 | 0.615 | **0.001** | 0.563 | 0.171 | 1.849 | 0.343 |
| Q16. Improves QoL | 0.343 | 0.175 | 0.669 | **0.002** | 0.629 | 0.191 | 2.070 | 0.445 |
| Q20. Control body weight | 0.554 | 0.318 | 0.964 | **0.037** | 0.505 | 0.135 | 1.896 | 0.312 |
| Q22. Enhances self-care abilities | 0.339 | 0.182 | 0.629 | **0.001** | 0.967 | 0.311 | 3.005 | 0.954 |
| Q23. Prevents other disease | 0.861 | 0.568 | 1.304 | 0.479 | 1.291 | 0.448 | 3.716 | 0.636 |
| **Barriers** | | | | | | | | |
| Q5. Tiredness | 1.790 | 1.189 | 2.696 | **0.005** | 0.706 | 0.230 | 2.167 | 0.543 |
| Q8. Adverse to health | 1.222 | 0.789 | 1.893 | 0.369 | 0.557 | 0.202 | 1.538 | 0.259 |
| Q9. Fear of falling | 2.129 | 1.404 | 3.227 | **<0.001** | 1.173 | 0.426 | 3.232 | 0.757 |
| Q11. Muscle fatigue | 1.693 | 1.124 | 2.548 | **0.012** | 1.124 | 0.405 | 3.120 | 0.822 |
| Q12. Lack of understanding of benefits | 1.851 | 1.124 | 3.047 | **0.015** | 1.778 | 0.541 | 5.843 | 0.343 |
| Q14. Other comorbidities | 3.389 | 2.065 | 5.561 | **<0.001** | 2.868 | 0.872 | 9.428 | 0.083 |
| Q15. Body pain | 1.853 | 1.234 | 2.785 | **0.003** | 2.088 | 0.732 | 5.956 | 0.169 |
| Q17. Lack of exercise knowledge | 1.943 | 1.199 | 3.148 | **0.007** | .992 | 0.336 | 2.924 | 0.988 |
| Q18. Worry about thirst | 0.949 | 0.634 | 1.419 | 0.797 | .893 | 0.311 | 2.562 | 0.833 |
| Q19. Have CKD | 1.918 | 1.165 | 3.159 | **0.010** | 1.087 | 0.351 | 3.372 | 0.885 |
| Q21. Worry affect arteriovenious fistula | 0.921 | 0.610 | 1.390 | 0.694 | 1.786 | 0.320 | 9.955 | 0.508 |
| Q24. Burden on family | 3.168 | 1.945 | 5.160 | **<0.001** | 1.571 | 0.510 | 4.837 | 0.431 |
| Note.  OR = Odds ratio: an OR above 1 denotes an increased likelihood of being physically inactive; an OR of less than 1 denotes a decreased likelihood of being inactive | | | | | | | | |

**Supplementary Material 7. The association between barriers and benefits to exercise and the likelihood of being physically inactive in younger and older patients (data used in Figure 3)**

| **Questions** | **Younger (≤65) (n=592)** | | | | **Older (>65) (n=554)** | | | |
| --- | --- | --- | --- | --- | --- | --- | --- | --- |
|  | **OR** | **Lower CI (95%)** | **Upper CI (95%)** | **P** | **OR** | **Lower CI (95%)** | **Upper CI (95%)** | **P** |
| **Benefits** | | | | | | | | |
| Q2. Reduces body pain | 0.689 | 0.424 | 1.118 | 0.131 | 0.362 | 0.168 | 0.780 | **0.010** |
| Q3. Postpones decline in body function | 0.663 | 0.365 | 1.204 | 0.177 | 0.122 | 0.029 | 0.512 | **0.004** |
| Q4. Prevents muscle atrophy | 0.942 | 0.541 | 1.640 | 0.832 | 0.289 | 0.101 | 0.828 | **0.021** |
| Q6. Improves mood | 0.560 | 0.299 | 1.048 | 0.070 | 0.288 | 0.119 | 0.697 | **0.006** |
| Q7. Improves bone disease | 0.731 | 0.425 | 1.258 | 0.258 | 0.567 | 0.264 | 1.215 | 0.144 |
| Q10. Improves appetite | 0.798 | 0.472 | 1.350 | 0.400 | 0.617 | 0.304 | 1.255 | 0.183 |
| Q13. Lead optimistic and active life | 0.583 | 0.304 | 1.118 | 0.104 | 0.164 | 0.050 | 0.539 | **0.003** |
| Q16. Improves QoL | 0.485 | 0.234 | 1.005 | 0.052 | 0.342 | 0.132 | 0.889 | **0.028** |
| Q20. Control body weight | 0.648 | 0.330 | 1.273 | 0.208 | 0.524 | 0.237 | 1.157 | 0.110 |
| Q22. Enhances self-care abilities | 0.530 | 0.283 | 0.995 | **0.048** | 0.253 | 0.089 | 0.721 | **0.010** |
| Q23. Prevents other disease | 0.803 | 0.492 | 1.312 | 0.381 | 1.087 | 0.575 | 2.053 | 0.798 |
| **Barriers** | | | | | | | | |
| Q5. Tiredness | 2.375 | 1.465 | 3.851 | **<0.001** | 0.943 | 0.485 | 1.832 | 0.862 |
| Q8. Adverse to health | 1.015 | 0.622 | 1.657 | 0.951 | 1.283 | 0.640 | 2.571 | 0.482 |
| Q9. Fear of falling | 1.820 | 1.129 | 2.933 | **0.014** | 2.410 | 1.240 | 4.684 | **0.009** |
| Q11. Muscle fatigue | 1.843 | 1.143 | 2.971 | **0.012** | 1.388 | 0.734 | 2.627 | 0.313 |
| Q12. Lack of understanding of benefits | 1.891 | 1.045 | 3.422 | **0.035** | 1.858 | 0.870 | 3.970 | 0.110 |
| Q14. Other comorbidities | 3.397 | 1.885 | 6.122 | **<0.001** | 3.004 | 1.443 | 6.253 | **0.003** |
| Q15. Body pain | 2.703 | 1.667 | 4.383 | **<0.001** | 1.219 | 0.652 | 2.279 | 0.536 |
| Q17. Lack of exercise knowledge | 2.034 | 1.140 | 3.637 | **0.016** | 1.331 | 0.667 | 2.617 | 0.407 |
| Q18. Worry about thirst | 0.996 | 0.622 | 1.597 | 0.988 | 1.049 | 0.556 | 1.977 | 0.884 |
| Q19. Have CKD | 1.757 | 0.950 | 3.247 | 0.072 | 1.548 | 0.775 | 3.095 | 0.216 |
| Q21. Worry affect arteriovenious fistula | 1.048 | 0.639 | 1.719 | 0.851 | 1.016 | 0.504 | 2.050 | 0.964 |
| Q24. Burden on family | 2.600 | 1.505 | 4.491 | **0.001** | 3.581 | 1.626 | 7.887 | **0.002** |
| Note.  OR = Odds ratio: an OR above 1 denotes an increased likelihood of being physically inactive; an OR of less than 1 denotes a decreased likelihood of being inactive | | | | | | | | |

**Supplementary Material 8. The association between age, gender, and haemoglobin levels and the likelihood of reporting the barrier or benefit to exercise**

| **Questions** | **Age** | | | | **Gender** | | | | **Haemoglobin (Hb)** | | | |
| --- | --- | --- | --- | --- | --- | --- | --- | --- | --- | --- | --- | --- |
|  | **OR** | **Lower CI (95%)** | **Upper CI (95%)** | **P** | **OR** | **Lower CI (95%)** | **Upper CI (95%)** | **P** | **OR** | **Lower CI (95%)** | **Upper CI (95%)** | **P** |
| **Benefits** | | | | | | | | | | | | |
| Q2. Reduces body pain | 0.998 | 0.988 | 1.008 | 0.663 | 0.782 | 0.573 | 1.067 | 0.121 | 1.069 | 0.962 | 1.188 | 0.213 |
| Q3. Postpones decline in body function | .996 | 0.984 | 1.007 | 0.476 | 0.895 | 0.620 | 1.291 | 0.552 | 0.992 | 0.876 | 1.124 | 0.902 |
| Q4. Prevents muscle atrophy | 0.989 | 0.977 | 1.000 | 0.058 | 0.961 | 0.668 | 1.382 | 0.828 | 1.046 | 0.925 | 1.183 | 0.474 |
| Q6. Improves mood | 0.982 | 0.972 | 0.993 | **0.002** | 0.961 | 0.681 | 1.356 | 0.820 | 1.039 | 0.923 | 1.168 | 0.528 |
| Q7. Improves bone disease | 0.998 | 0.987 | 1.009 | 0.731 | 0.983 | 0.875 | 1.104 | 0.774 | 1.024 | 0.724 | 1.448 | 0.894 |
| Q10. Improves appetite | 0.986 | 0.975 | 0.997 | **0.011** | 0.727 | 0.521 | 1.013 | 0.060 | 1.021 | 0.911 | 1.145 | 0.721 |
| Q13. Lead optimistic and active life | 0.984 | 0.972 | 0.995 | **0.006** | 0.913 | 0.637 | 1.308 | 0.619 | 0.976 | 0.863 | 1.105 | 0.706 |
| Q16. Improves QoL | 0.979 | 0.967 | 0.992 | **0.001** | 0.946 | 0.646 | 1.385 | 0.776 | 1.041 | 0.913 | 1.188 | 0.546 |
| Q20. Control body weight | 0.980 | 0.969 | 0.993 | **0.002** | 0.720 | 0.500 | 1.036 | 0.077 | 0.987 | 0.870 | 1.121 | 0.841 |
| Q22. Enhances self-care abilities | 0.998 | 0.987 | 1.009 | 0.685 | 0.895 | 0.629 | 1.273 | 0.538 | 1.052 | 0.934 | 1.185 | 0.406 |
| Q23. Prevents other disease | 1.002 | 0.993 | 1.012 | 0.627 | 0.637 | 0.465 | 0.873 | **0.005** | 1.051 | 0.945 | 1.170 | 0.361 |
| **Barriers** | | | | | | | | | | | | |
| Q5. Tiredness | 0.992 | 0.981 | 1.002 | 0.106 | 1.1190 | 0.806 | 1.554 | 0.503 | 0.913 | 0.817 | 1.020 | 0.106 |
| Q8. Adverse to health | 0.991 | 0.981 | 1.001 | 0.083 | 1.121 | 0.806 | 1.561 | 0.497 | 0.932 | 0.834 | 1.041 | 0.213 |
| Q9. Fear of falling | 1.005 | 0.995 | 1.014 | 0.340 | 1.392 | 1.021 | 1.896 | **0.036** | 1.021 | 0.920 | 1.132 | 0.701 |
| Q11. Muscle fatigue | 0.997 | 0.987 | 1.007 | 0.579 | 1.036 | 0.743 | 1.444 | 0.836 | 0.841 | 0.751 | 0.942 | **0.003** |
| Q12. Lack of understanding of benefits | 1.013 | 1.002 | 1.024 | **0.017** | 1.186 | 0.847 | 1.661 | 0.321 | 0.964 | 0.859 | 1.082 | 0.534 |
| Q14. Other comorbidities | 1.018 | 1.008 | 1.029 | **<0.001** | 1.090 | 0.798 | 1.490 | 0.588 | 0.975 | 0.877 | 1.085 | 0.648 |
| Q15. Body pain | 0.996 | 0.986 | 1.006 | 0.396 | 1.030 | 0.756 | 1.405 | 0.850 | 0.943 | 0.849 | 1.048 | 0.275 |
| Q17. Lack of exercise knowledge | 1.009 | 0.998 | 1.020 | 0.094 | 1.307 | 0.941 | 1.816 | 0.110 | 0.914 | 0.816 | 1.023 | 0.118 |
| Q18. Worry about thirst | 0.986 | 0.976 | 0.996 | **0.005** | 1.137 | 0.832 | 1.554 | 0.420 | 0.972 | 0.876 | 1.079 | 0.595 |
| Q19. Have CKD | 1.017 | 1.006 | 1.029 | **0.002** | 1.187 | 0.849 | 1.661 | 0.316 | 0.953 | 0.849 | 1.069 | 0.409 |
| Q21. Worry affect arteriovenious fistula | 0.985 | 0.975 | 0.995 | **0.003** | 0.784 | 0.563 | 1.090 | 0.147 | 0.917 | 0.822 | 1.023 | 0.120 |
| Q24. Burden on family | 1.006 | 0.996 | 1.016 | 0.223 | 1.450 | 1.059 | 1.985 | **0.021** | 1.009 | 0.908 | 1.122 | 0.861 |
| Note.  OR = Odds ratio: an OR above 1 denotes an increased likelihood of reporting the barrier or benefit; an OR of less than 1 denotes a decreased likelihood of reporting the barrier or benefit | | | | | | | | | | | | |
